# Design of Lamifuse: a randomised, multi-centre controlled trial comparing laminectomy without or with dorsal fusion for cervical myeloradiculopathy

**DOI:** 10.1186/1471-2474-8-111

**Published:** 2007-11-09

**Authors:** Ronald HMA Bartels, André LM Verbeek, J André Grotenhuis

**Affiliations:** 1 Department of Neurosurgery, Radboud University Nijmegen Medical Centre, Nijmegen, The Netherlands; 2 Department of Biostatistics and Epidemiology, Radboud University Nijmegen Medical Centre, Nijmegen, The Netherlands

## Abstract

**Background:**

laminectomy is a valuable surgical treatment for some patients with a cervical radiculomyelopathy due to cervical spinal stenosis. More recently attention has been given to motion of the spinal cord over spondylotic spurs as a cause of myelopathic changes. Immobilisation by fusion could have a positive effect on the recovery of myelopathic signs or changes. This has never been investigated in a prospective, randomised trial. Lamifuse is an acronyme for laminectomy and fusion.

**Methods/Design:**

Lamifuse is a multicentre, randomised controlled trial comparing laminectomy with and without fusion in patients with a symptomatic cervical canal stenosis. The study population will be enrolled from patients that are 60 years or older with myelopathic signs and/or symptoms due to a cervical canal stenosis. A kyphotis shape of the cervical spine is an exclusion criterium. Each treatment arm needs 30 patients.

**Discussion:**

This study will contribute to the discussion whether additional fusion after a cervical laminectomy results in a better clinical outcome.

**ISRCT number:**

ISRCTN72800446

## Background

Cervical spondylosis is a progressive degenerative disease of the spine. As people grow older, the prevalence of cervical spondylosis increases. It is a natural process of aging. Cervical spondylosis is seen in 10% of individuals in the age of 25 years, whereas in 95% of the persons of 65 years [1].

Due to the degenerative process reduction of height of the intervertebral discs, formation of spondylophytes and sometimes instability occurs. This may lead to a stenosis of the cervical spinal canal. In most instances it will remain asymptomatic. However, in some persons the stenosis of the spinal canal leads to a compression of the spinal cord. It is important to realize that not only static compression leads to neurological symptoms, but also dynamic factors do. In a normal situation the spinal cord will move during flexion and extension. Ventral osteophytes in the spinal canal prevent up – and downward movement [1]. Furthermore, the spinal cord is more stretched over the anterior bars increasing axial tension within the spinal cord. These forces are multidirectional creating secondary shearing forces resulting in stretch and shear injury to myelin and neural elements [2-4].

Patients may present with a diversity of well known signs and symptoms with variable intensities. Disturbance of the sensibility in the arms, clumsiness of the hands and problems with micturation may occur. However, the hallmark symptoms are gait abnormalities, weakness of the legs or stiffness of the legs [1,5].

The natural course of the cervical myelopathy is variable. But patients developing mild or moderate symptoms are less likely to improve spontaneously. Non operative treatment will mainly affect neck pain or accompanying radiculopathy. Improvement has been noted but is variable [6,7]. Patients with myelopathic signs and symptoms will, however, likely benefit from surgery [5,7,8].

Surgical approaches for cervical myelopathy due to cervical spondylosis can be anterior, posterior or combined. The last option is reserved for deformity correction. In most instances a lordotic or slight kyphotic cervical spine is present. The choice for an anterior or posterior approach is dependent on the main site of compression, the shape of the cervical sagittal curvature and to a lesser account on the preference of the surgeon.

Dorsal approaches are laminectomy or laminoplasty. A difference in clinical outcome has never been established. Prevention of post – laminectomy kyphosis is a reason for laminoplasty. If an additional, instrumented dorsal fusion is performed, the change of developing a post-surgical kyphosis is nearly zero[9]. It should be memorized that spondylotic processes also generate reduced motion of the spinal segments, a natural course [1]. From this point of view, decompression with fusion will have better clinical results when compared to decompression solely. In literature, indications in this direction are found. Frequently used dorsal fusion techniques today use lateral mass screws and cervical pedicle screws. This is relatively safe with a minimal persistent complication rate. Furthermore, in experienced hands these techniques do not add substantial time to the duration of the surgery [7,9].

Despite a long-lasting interest in the various techniques, the clinical superiority of one method over the other has never been established. To our knowledge, a randomized controlled trial comparing laminectomy with or without fusion has never been performed.

## Methods/Design

### Hypothesis

Patients that are surgically treated for signs and symptoms due to a stenosis of the cervical spinal canal have a better clinical outcome when a dorsal fusion is performed in addition to a laminectomy compared to those that have solely a laminectomy.

At the end of the study, the quality of life, complications, and the costs will be evaluated comparing these two treatment groups.

### In – and exclusion criteria

**Figure 1:**
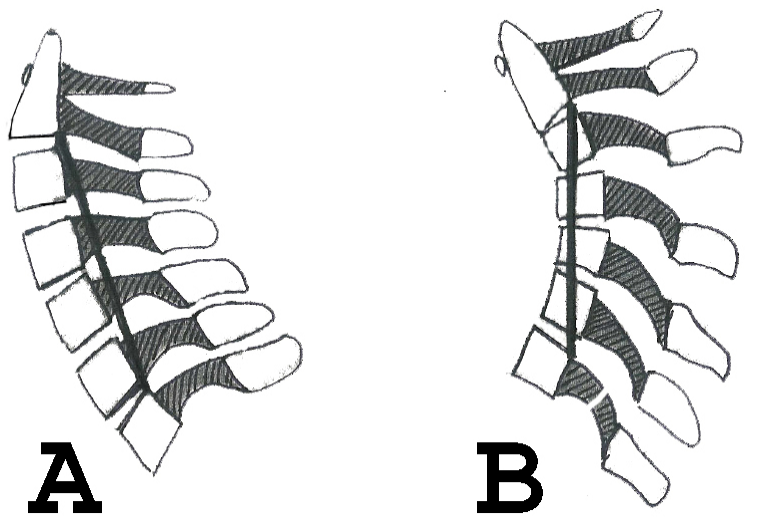
Line from the posterior inferior part of the vertebral body of C2 to the posterior superior part o f the vertebral body of C7 in case of a normal cervical lordotic curvature (A) and a kyphotic cervical curve (B).

Patients with a minimal age of 60 years are included (Table 1). At neurologic examination myelopathic changes must be apparent. At magnetic resonance imaging, concordant stenotic alterations at the cervical level(s) must be present. At the plain sitting lateral radiograph a lordotic spine must be shown. The shape of the cervical spine is lordotic when the vertebral bodies of C3 to C6 are in front of a line drawn from a point of the posterior inferior part of the vertebral body of C2 to a point at the posterior superior part of the vertebral body of C7 (Figure 1).

**Table 1:** in – and exclusioncriteria

Inclusion	Exclusion
60 years or older	Previous cervical surgery for myelopathic signs and symptoms
Cervical myelopathic symptoms and or signs at neurologic examination	Solely radiculopathy, or most important complaint
Stenosis of cervical spinal canal at MRI	Unable to undergo MRI
Lordotic shape at lateral cervical plain radiograph, or at lateral cervical radiograph in extension	Life expectancy less than 2 years
Informed consent	Other diseases interfering with neurologic symptoms and signs, for example spinal cord glioma, thoracic herniated disc with spinal cord compression, multiple sclerosis etc.
	Rheumatoid arthritis
	Trauma to the neck in history
	Diseases interfering with rehabilitation, for example severe cardiac congestive disease.
	Participation in another study

Only patients that sign the informed consent after some time of reflection (1 week) are included.

### Clinical evaluation and follow up

At the first intake, duration of symptoms, other diseases, severity of signs and symptoms are noted. Neurologic examination is performed by an independent neurologist. At follow up, the severity of signs and symptoms are also noted. Special attention is given to the myelopathic changes in arms and/or legs. Furthermore, the clinical situation is evaluated by the modified Japanese Orthopaedic Association functional score[10] translated into the Dutch language. The validation of the Dutch translation is currently subject of investigation. Finally, a change in the quality of life is evaluated by the Dutch Short Form – 36 Health Survey.

Follow – up will be at six weeks, six months, and after one year postoperatively (Table 2). Complications are noted for their nature, duration and severity

**Table 2:** overview of investigations at each clinical contact

	Preoperative	Postoperative (po)	6 weeks po	3 months po	1 year po
MRI	**X**				
Plain cervical radiograph	**X**	**X**	**X**	**X**	**X**
mJOA	**X**		**X**	**X**	**X**
SF-36	**X**		**X**	**X**	**X**
Neurologic examination by independent neurologist	**X**		**X**		**X**

### Surgical technique

Cervical laminectomy of the compressed levels is performed. Previous to the laminectomy a dorsal fusion is done. Dorsal fusion includes lateral mass screws from C2 to C6. In C2, C7 and the upper thoracic spine levels, pedicle screws will be placed. The screws are connected by rods or plates. Transverse connectors are used when indicated. In order to keep the posterior tension band intact, the fusion will extend from one level above the planned most cranial laminectomy level to at least one level below the most caudal planned laminectomy site. If the lowest level of fusion would include C7 or lower extension of the fusion to the upper thoracic spine (Th2 or Th3) is recommended[11]. This extension of the fusion is thought to prevent junction disease at the cervicothoracic junction. For example, if the laminectomy includes the levels C4 to C6, the fusion would be from C3 to C7. Because C7 is the lowest fusion level, incorporation of Th1 is recommended.

### Surgical demands

Since fusion is added, only centres with a known spinal surgical experience are asked to participate. The surgeons performing a laminectomy should also be experienced in lateral mass fixation techniques, especially lateral mass and cervical pedicle screws.

### Study Sites

The following centres will paricipate: Radboud University Nijmegen Medical Centre, Nijmegen; Canisius Wilhelmina Hospital, Nijmegen; Medical Centre Haaglanden, The Hague; Sint Maartens Hospital, Nijmegen. All Centres are located in the Netherlands. In all centres ethical approval is obtained.

### Statistical analysis

The primary endpoint is clinical outcome after 1 year using the modified Japanese Orthopaedic Association functional score. Secondary outcomes are cost-effectiveness, quality of life measured by the SF-36, and complications. Statistical analysis is performed by a blinded investigator. For Statistical analysis the SAS system is used. Descriptive statistics are used to describe baseline characteristics. For comparison between groups student-t tests or chi – square tests are used. Statistical significance is reached when p < 0.05. Risk ratios (RR) and 95 % confidence limits (CI) are presented. All analyses are done according to the intention – to – treat principle

The minimal clinically important difference was estimated by asking 4 international active spine surgeons what they would consider a clinically significant difference in mJOA score. The mean of the values is considered the MCID.

The sample size is calculated as follows: a difference of at least two points on the modified JOA functional score is considered significant. The difference is expected to be mainly allocated to the function of the arms and legs. Based on literature, a standard deviation of approximately 2 is assumed [12]. A two group student t test with a 0.05 two sided significance level and a power of 95 % will need a sample size per group of 27 to detect a significant difference. Considering a ten percent of lost to follow up, a total of 30 individuals per group will be included.

### Randomisation

For randomisation, the closed envelope method is used. As soon as informed consent is obtained, one of the treatment options is assigned to the patient. The secretary of the neurosurgical department in the Canisius Wilhelmina Hospital, Nijmegen, the Netherlands will control the randomisation. Prior to surgery, the patient is informed about the chosen option. Patients who do not choose for participation, are offered one of the surgical options that are currently under investigation. However, they are not followed in an observational cohort study.

### Endpoints

#### Primary endpoints

**Figure 2:**
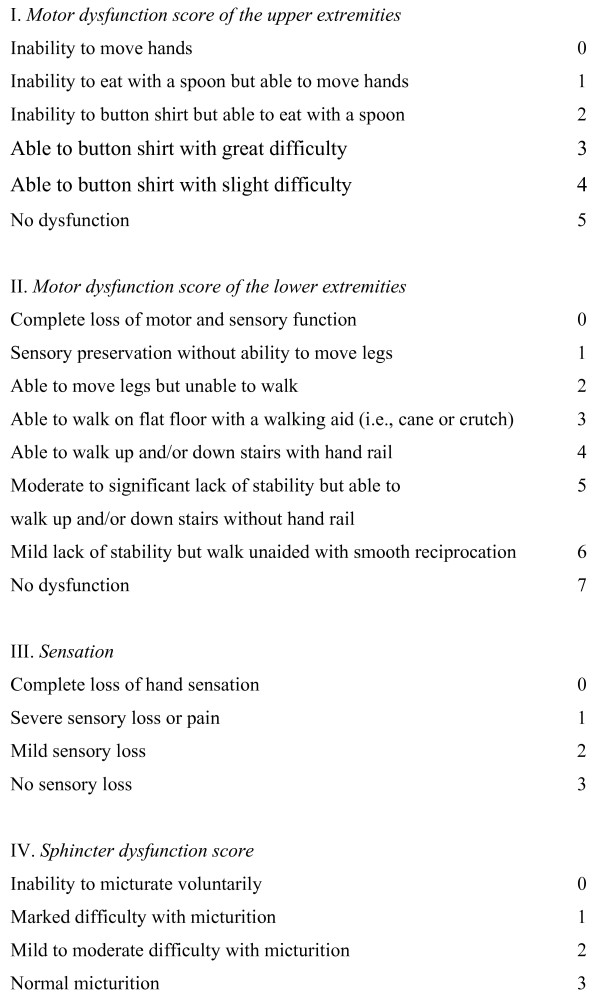
Modified Japanese Orthopaedic Association functional score.

Several score systems exist for grading the severity of cervical myelopathy. The modified Japanese Orthopedic functional score (Fig. 2) evaluates four groups: the function of the arms, of the legs, the micturation, and the sensibility of the hands. It has the major advantage that it assesses these functions separately [10]. Although it has been established that outcome after decompressive surgery reaches a plateau at six months postoperatively [13], the primary endpoint will be evaluated at one year postoperatively just to make sure.

#### Secondary endpoints

Since instrumentation is added in the fusion group, the costs will be higher. On the other hand, it is assumed that a mean better recovery will take place in the fusion group. Therefore, the additional costs (nursing costs, auxillary supports, etc.) may be lower. A careful evaluation of the costs of the treatment related to the outcome is performed. To obtain a reliable insight in the costs the following will be noted in a kind of diary: hospitalisation, out – patient contacts, additional medication, house keeping support, instruments to support daily activities, e.g. walking, eating etcetera. Of each item the sort and amount will be recorded.

Apart from the cost – effectiveness, the difference in the general health status will be evaluated. It is assumed that general health will improve more after of a laminectomy with a fusion than one without. This will be reflected in a difference of the SF – 36 score. The Short Form 36 Health Status Questionnaire is a widely-used generic health status. This instrument consists of eight subscales and two summary scales. On each scale higher scores indicate better outcomes. Scores can be compared with published age – and sex – matched general population or disease-specific norms [14]. Furthermore, it has been validated for cervical spondylotic myelopathy [15].

Complications are separately registered. Complications related to the cervical myelopathy are postoperative hemorrhage, postoperative infection, temporary or permanent impairment of neurologic function, and kyphotic deformation of the cervical spine[7]. Complications related to adding lateral mass screws or/and pedicle screws are vertebral artery injury and temporary or permanent nerve root damage[7]. In order to prevent damage to the spinal cord, the instrumentation should be completed before the laminectomy.

### Monitored events

Monitored events are the death of a patient, withdrawal from the study, lost to follow – up, and cross – over from their randomly assigned treatment group. These events are registered within the case record form. The circumstances of the events are investigated and also noted. In case of death of the patient, a search for a relationship with the instituted treatment is started. Throughout the study, all medical complications and intervening treatments concerning the cervical spine are registered within the CRF at the usual follow – up visits or when the appropriate information reaches the treating surgeon.

### Protocol violations

Any of the following will be considered as a deviation from the protocol: randomization of an ineligible patient, enrollment of a patient that is already participating in an another study, enrollment of an participant to this study in another study, a patient receiving the wrong treatment, loss of radiology or any other data, and informed consent violations. Violations are reviewed monthly and reported to the independent study supervisor (R.D. Donk, M.D., Canisius Wilhelmina Hospital, Nijmegen).

### Subject confidentiality

The anonymity of the subjects will be maintained. Subjects will be identified by their initials and a subject number assigned by the secretary of the neurosurgical department in the Canisius Wilhelmina Hospital, Nijmegen, the Netherlands. All CRFs and other documents submitted to the investigator will be assigned this code. The secretary will enter the data of the patient and their assigned code. This list is only accessible for the principal investigator, and the independent supervisor.

## Discussion

Movement as an cause additional to stenosis of cervical myelopathy has been recognised for longer time. A positive effect of fusion in addition to decompression by laminectomy has been reported earlier. To our knowledge, the is the first randomised controlled trial comparing laminectomy without and with dorsal instrumented fusion. Apart from the clinical effectiveness, a study is needed to explore the costeffectiveness of the treatments.

Several problems may arise. Randomization may be refused by some patients. However, experience from earlier trials learned that after correct description of the possibilities and estimated outcomes patients will not be reluctant to be enrolled.

Through the age restriction, the results of the study cannot be generalized to the whole population. However, most often the patients with a symptomatic, degenerative, cervical spinal canal stenosis will be 60 years old or older [16].

Since knowledge of the instrumentation and of biomechanics of the cervical spine is necessary to avoid complications and to optimise instrumented fusion only experienced spinal surgeons will collaborate with this study. This will prevent a discussion related to the presumed different skill levels of the surgeons in case of an unexpected result.

Finally, as a measure for the definitive clinical result the mJOA is chosen. This is the only scale that takes the ambulatory function, the function of the hands, the sensibility of the hands, and the micturation pattern separately into account. This scale is validated for the Japanese population [17]. However, its English translation has never been validated. Now, the English mJOA has been translated into Dutch. The validation of this translation is currently under investigation.

Finally, Lamifuse tries to measure a difference in costs. Since most of the patient will not be working anymore but are retired, costs of material or personal support, extra medication etcetera will be calculated. These costs are specific for the Netherlands, and cannot be extrapolated to other countries.

## Conclusion

Laminectomy for symptomatic cervical spinal stenisos is frequently performed. However, not only compressive forces are responsible for the complaints of the patient. Continuing motion of the spinal cord over anterior spondylophytic ridges is also believed to be a causative factor. Fusion will prevent motion. This randomised, controlled study will compare the clinical results of laminectomy without and with fusion for patients with a symptomatic stenosis of the cervical spinal canal. The design of Lamifuse is discussed as are its limitations.

## Competing interests

The author(s) declare that they have no competing interests.

## Authors' contributions

R.B. Generating the idea, writing the manuscript, epidemiological background

A.V. epidemiological background, revising manuscript

J.A. revising manuscript

## Pre-publication history

The pre-publication history for this paper can be accessed here:


